# Effects of ‘Healthy’ Fecal Microbiota Transplantation against the Deterioration of Depression in Fawn-Hooded Rats

**DOI:** 10.1128/msystems.00218-22

**Published:** 2022-04-28

**Authors:** Bing Hu, Promi Das, Xianglin Lv, Meng Shi, Jiye Aa, Kun Wang, Liping Duan, Jack A. Gilbert, Yong Nie, Xiao-Lei Wu

**Affiliations:** a College of Engineering, Beijing University, Beijing, People’s Republic of China; b Institute of Biochemical Engineering, College of Chemistry and Chemical Engineering, Beijing Institute of Technology, Beijing, People’s Republic of China; c Department of Pediatrics and Scripps Institution of Oceanography, University of California San Diego, La Jolla, California, USA; d Beijing University Hospital of Stomatology, Beijing, People’s Republic of China; e Key Lab of Drug Metabolism and Pharmacokinetics, China Pharmaceutical University, Nanjing, People’s Republic of China; f Beijing University Third Hospital, Beijing, People’s Republic of China; g Institute of Ecology, Beijing University, Beijing, People’s Republic of China; Istanbul Medipol University School of Medicine

**Keywords:** depression aggravation, fecal microbiota transplantation, metabolomics, microbiota-gut-brain (MGB) axis, neuromodulation

## Abstract

Depression is a recurrent, heterogeneous mood disorder occurring in more than 260 million people worldwide. Gut microbiome dysbiosis is associated with the development of depressive-like behaviors by modulating neuro-biochemical metabolism through the microbiome-gut-brain (MGB) axis. Fecal microbiota transplantation (FMT) has been proposed as a potential therapeutic solution for depression, but the therapeutic efficiency and mechanism are unknown. Here, we performed an FMT from Sprague-Dawley (SD) rats (‘healthy’ controls) to Fawn-hooded (FH) rats (depression model). Pre-FMT, the FH rats exhibited significantly elevated depressive-like behaviors and distinct neurotransmitter and cytokine levels compared with SD rats. Post-FMT, FH recipients receiving FH fecal microbiota (FH-FH rats) showed aggravated depressive-like behaviors, while the ones receiving SD microbiota (FH-SD rats) had significantly alleviated depressive symptoms, a significant increase in hippocampal neurotransmitters, and a significant decrease of some hippocampal cytokines than FH-FH rats. SD-FMT resulted in the FH-SD rats’ gut microbiome resembling the SD donors, and a significant shift in the serum metabolome but not the hippocampal metabolome. Co-occurrence analysis suggests that SD-FMT prevented recipients’ depression development via the significant decrease of gut microbial species such as *Dialister* sp., which led to the recipients’ metabolic modulation in serum and hippocampus through the enteric nervous system, the intestinal barrier, and the blood-brain barrier. Our results provided new data pointing to multiple mechanisms of interaction for the impact of gut microbiome modulation on depression therapy.

**IMPORTANCE** Depression is a chronic, recurrent mental disease, which could make the patients commit suicide in severe cases. Considering that gut microbiome dysbiosis could cause depressive symptoms in animals through the MGB axis, the modification of gut microbiota is expected to be a potential therapy for depression, but the daily administration of probiotics is invalid or transient. In this study, we demonstrated that the gut microbiome transferred from a healthy rat model to a depressive rat model could regulate the recipient’s neurobiology and behavior via the systematic alternation of the depressive gut microbiota followed by the serum and hippocampal metabolism. These results underline the significance of understanding the impact of gut microbiota on mental disorders and suggest that ‘healthy’ microbiota transplantation with the function to solve the host’s cerebral inflammation may serve as a novel therapeutic strategy for depression.

## INTRODUCTION

Depression is a recurrent, heterogeneous mood disorder, occurring in more than 260 million people worldwide (https://www.who.int/news/item/02-08-2021-fifa-launches-reachout-campaign-for-better-mental-health). Its etiology involves impaired regulatory mechanisms of neuroendocrine, immune, and neurotransmitter systems (1). Although there has been little progress in the identification of biomarkers, much of the research converges on the decreased concentration of monoamine neurotransmitters (e.g., serotonin and noradrenaline) in the brain, the atrophy of mature neurons in the hippocampus, or reduced neurogenesis in the hippocampus (2). Clinical studies have also demonstrated that, compared with the healthy persons, the chronically depressed patients had significantly increased proinflammatory cytokines, such as tumor necrosis factor (TNF)-α, interleukin (IL)-1β, and IL-6, which were immune communicators between the brain and the peripheral system (3). This suggests that depressive disorders were associated with long-term impairment of neuroinflammatory molecules in the brain and blood.

Numerous studies have provided evidence suggesting that the gut microbiome and the associated metabolites play important roles in modulating neuro-biochemistry and behaviors through the microbiota-gut-brain (MGB) axis (4–6). Studies in humans (7–9), macaques (10), and rodents (11) have demonstrated a variable microbiota signature associated with major depressive disorder or similar depression-like phenotypes. The gut microbiome can also be used to transfer this phenotype between hosts, such that germ-free mice or antibiotic-treated rats exhibited depressive-like behaviors 2 weeks after they have been colonized with the fecal microbiota of depressed human patients (12, 13).

Pioneering studies examining the relationship between the gut microbiota and major depressive disorder have suggested that a disrupted microbiome (dysbiosis) can directly increase intestinal permeability, thereby inducing systematic inflammation by translocation of immune activators (13–18). This immune activation may also trigger an alteration in neurotransmitter production (19–21), influencing brain activity and resulting in central nervous system (CNS) disorders (22). However, the mechanisms remain elusive, and a lack of identified causality hinders the translation of preventive and therapeutic strategies to the clinic.

Despite the lack of validated causal mechanisms, gut microbiome modulation has already been proposed as a potential therapeutic solution for depression (23, 24). Administration of the probiotic Bifidobacterium longum NCC3001 reduced both symptoms of irritable bowel syndrome (IBS) and depression in patients, potentially due to the reduction in limbic reactivity (25). Daily administration of Faecalibacterium prausnitzii ATCC 27766 for 4 weeks significantly alleviated anxiety- and depressive-like behaviors in rats, possibly via the regulation of host cytokine metabolism (26). However, any beneficial effect of probiotic therapy might be transient. For instance, the anxiolytic and antidepressant-like effects of probiotic therapy lasted no more than 2 months in older adults (27). This is likely driven by the resilience of the indigenous gut microbiota to the invasion of new species. Therefore, it is suggested that current psychobiotics may have limited potential to disrupt a depression-associated gut microbial ecosystem unless more complex communities are used (28). Manipulating the gut-brain axis of depression via fecal microbiota transplantation (FMT) has been explored by using the fecal matter of depressed donors in germ-free mice (29, 30), but whether FMT from a ‘healthy’ donor can influence recipient depressive symptoms has yet to be explored.

Here, the impact of microbiome transfer from a conventional animal model, Sprague Dawley (SD) rats, to a genetically impaired animal model, Fawn-hooded (FH) rats, which are known for their altered serotonergic activity (31), on the recipient’s neurophysiology, immune profile, microbiome, and behavior was determined. This model system was used to test the following hypotheses: (i) FMT would result in increased microbiome similarity between the recipient and the donor; (ii) the recipient’s neurobehavioral, physiochemical, and immunological characteristics would be within the quantified ranges demonstrated by the donor following FMT. To test these hypotheses, fecal, serum and hippocampal tissue samples were collected from the experimental rat groups to characterize the fecal microbiome by metagenomics and to quantify the microbial metabolites in serum and hippocampal tissue by untargeted metabolomics and immunoassays. It was demonstrated that FMT from the ‘healthy’ SD rats to the ‘depressed’ FH rats resulted in significant changes in the microbiome, serum metabolome, hippocampal cytokine profiles and neurotransmitter levels, and behaviors in the FH recipients.

## RESULTS

### Characterization of neurobehaviors and depression-associated neurotransmitters and cytokines in the control and FMT-processed rats.

**FIG 1:**
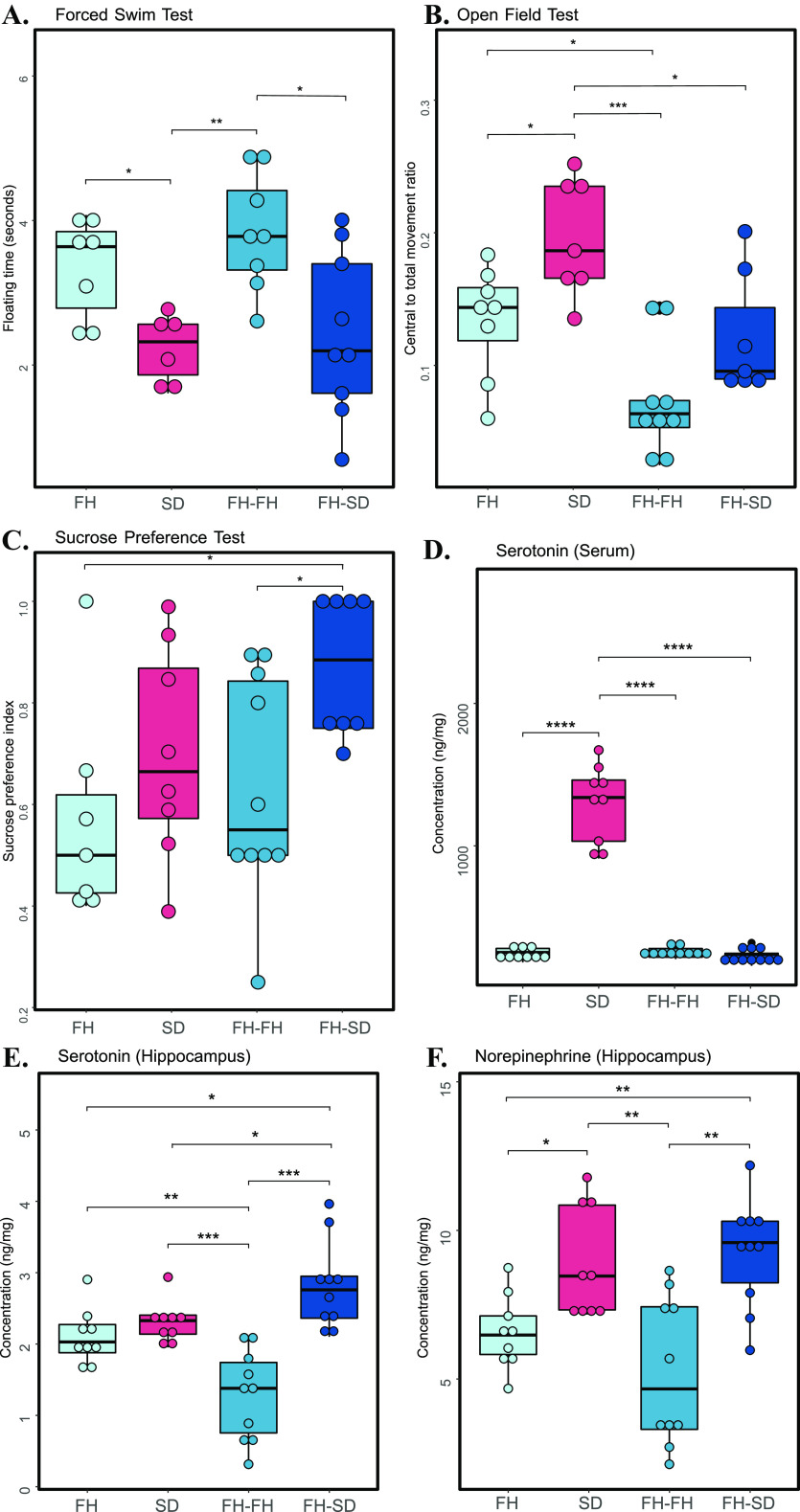
Physiological characterization of neurobehavioral and neurotransmitters. Boxplots showing the test scores of forced swim test (A), open field test (B), sucrose preference test (C), the concentration of serotonin in serum (D), and concentrations of serotonin and norepinephrine in the hippocampus (E to F). The asterisks indicate as follows: ns, adjusted *P* ≥ 0.05; *, adjusted *P* < 0.05; **, adjusted *P* < 0.01; ***, adjusted *P* < 0.001; ****, adjusted *P* < 0.0001 (Student's *t* test for FST, OFT, SPT, hippocampal serotonin and norepinephrine; Wilcoxon test for serum serotonin).

To test if the neurobehavioral characteristics of the FH recipients upon SD- and FH-FMT were differentially altered, three anxiety-like and depressive-like behavioral tests, including the forced swim test (FST), open-field test (OFT), and sucrose preference test (SPT), were conducted on the two groups of FMT-processed FH rats and the control SD and FH rats. Significant differences were observed among the four groups of rats for FST (one-way ANOVA, adjusted *P* = 0.004), OFT (one-way ANOVA, adjusted *P* = 0.00012), and SPT (one-way ANOVA, adjusted *P* = 0.031). As shown in Fig. 1A to C, compared with the healthy SD rats, FH rats displayed a significant increase in the immobility time during FST (two-sample unpaired *t* test, adjusted *P* = 0.020), a significant decrease in the central to total movement ratios during OFT (two-sample unpaired *t* test, adjusted *P* = 0.025), and a nonsignificant decrease in sucrose preference index during SPT (two-sample unpaired *t* test, adjusted *P* = 0.338). Interestingly, FH recipients receiving FH fecal microbiota (FH-FH rats), compared with FH rats, displayed further higher differences with SD rats on the FST scores (two-sample unpaired *t* test, adjusted *P* = 0.007; Fig. 1A) and OFT scores (two-sample unpaired *t* test, adjusted *P* = 0.0005; Fig. 1B), indicating that the ‘depressive’ microbiota transplantation to FH rats could lead to the aggravation of depressive-like behaviors. Similar to the trends of differences between FH and SD rats, FH-FH rats had significantly higher FST scores (two-sample unpaired *t* test, adjusted *P* = 0.025; Fig. 1A), insignificantly lower OFT scores (two-sample unpaired *t* test, adjusted *P* = 0.066, Fig. 1B), and significantly lower SPT scores (two-sample unpaired *t* test, adjusted *P* = 0.047, Fig. 1C) than FH recipients receiving SD fecal microbiota (FH-SD rats). Except for that in OFT, FH-SD rats had similar levels of FST scores (two-sample unpaired *t* test, adjusted *P* = 0.701; Fig. 1A) and SPT scores (two-sample unpaired *t* test, adjusted *P* = 0.184; Fig. 1C) with SD rats. Altogether, these results indicated that the ‘healthy’ microbiota transplantation to FH rats could prevent the development of FH-SD rats’ depressive symptoms.

Similarly, to determine if the depression-related neurotransmitter concentrations altered in FH-FH and FH-SD rats, we measured two major neurotransmitters (i.e., serotonin and norepinephrine) in all four groups of rats. Owing to the genetic dysfunctional nature of the serotoninergic system in the FH rats, the serotonin concentrations in serum of FH rats (unpaired Wilcoxon test, adjusted *P* = 8.22 × 10^−5^), FH-FH rats (unpaired Wilcoxon test, adjusted *P* = 6.51 × 10^−5^) and FH-SD rats (Wilcoxon test, adjusted *P* = 6.51 × 10^−5^) were significantly lower than those in SD rats’ serum (Fig. 1D). Nevertheless, the contents of hippocampal serotonin were significantly different between the four groups of rats (one-way ANOVA, adjusted *P* = 1.2 × 10^−6^). As shown in Fig. 1E, FH rats had insignificantly lower content of hippocampal serotonin than SD rats (two-sample unpaired *t* test, adjusted *P* = 0.180). The contents of hippocampal serotonin in FH-FH rats were significantly lower than those in SD rats (two-sample unpaired *t* test, adjusted *P* = 0.0008), suggesting that the hippocampal serotonin metabolism in FH-FH rats was abnormal. However, the reduction was inferred to be remedied by the ‘healthy’ fecal microbiota, because the contents of hippocampal serotonin in FH-SD rats were observed to be significantly higher than those in FH-FH rats (two-sample unpaired *t* test, adjusted *P* = 0.0002) and FH rats (two-sample unpaired *t* test, adjusted *P* = 0.011). Similarly, the variations of hippocampal norepinephrine contents between the four groups were also significant (one-way ANOVA, adjusted *P* = 0.0002), and their pairwise comparisons were shown in Fig. 1F. It was observed that the contents of hippocampal norepinephrine in SD rats were significantly higher than those in FH rats (two-sample unpaired *t* test, adjusted *P* = 0.014) and FH-FH rats (two-sample unpaired *t* test, adjusted *P* = 0.006). The contents in FH-SD rats were also significantly higher than those in FH rats (two-sample unpaired t test, adjusted *P* = 0.005) and FH-FH rats (two-sample unpaired *t* test, adjusted *P* = 0.004), while they were similar to those in SD rats (two-sample unpaired *t* test, adjusted *P* = 0.574). One plausible inference of these observations could point out that the transplantation of SD fecal microbiota in FH rats influenced their neuromodulation in the hippocampus through the enteric nervous system (ENS), but not the neurotransmitter metabolism in serum through the intestinal barrier. Interestingly, the more obvious differences between FH-SD and FH-FH rats than those between SD and FH rats for the hippocampal serotonin suggested some unknown biological effect of FMT on the neurotransmitter pool in the hippocampus.

To determine if FMT influenced immune responses in the rats, cytokines in both the serum and hippocampus of the control and FMT-processed rats were quantified. It was found that the contents of serum cytokines IL-2, IL-4, IL-6, IL-10, IL-17A, and interferon (IFN)-γ were significantly different between the four groups of rats (Kruskal-Wallis test, adjusted *P* < 0.05), while the levels of serum IL-1β and TNF-α were not very different among the four groups (Kruskal-Wallis test, adjusted *P* ≥ 0.05). As shown in Fig. S1 (left panel), compared with those in FH rats, the contents of serum IL-2, IL-6, and IL-17A were significantly lower in FH-FH rats, while the levels of serum IL-4, IL-10, and IFN-γ in FH-FH rats were significantly higher in FH-FH rats (Wilcoxon test, adjusted *P* < 0.05). The increasing or reduction of serum cytokines in FH-FH rats was not suppressed by SD-FMT processing, because the contents of these serum cytokines in FH-FH and FH-SD rats had no significant differences (Wilcoxon test, adjusted *P* ≥ 0.05). However, considering that, all measured serum cytokines had similar contents between FH and SD rats in Fig. S1 (Wilcoxon test, adjusted *P* > 0.05), it was inferred that serum cytokine metabolism and the depression of FH rats had no causal links. For the hippocampal cytokines, the levels of hippocampal cytokines IL-1β (two-sample unpaired *t* test, adjusted *P*_IL-1β_ = 0.047) and TNF-α (Wilcoxon test, adjusted *P*_TNF-α_ = 0.012) were significantly lower in SD rats than those in FH rats, respectively, and they also showed trends toward lower levels in FH-SD rats compared to FH-FH rats (two-sample unpaired *t* test, adjusted *P*_IL-1β_ = 0.16; Wilcoxon test, adjusted *P*_TNF-α_ = 0.021; Fig. S1 right panel). Interestingly, hippocampal IL-17A contents in FH-FH rats were significantly higher than that in FH (two-sample unpaired *t* test, *P* = 0.027) and FH-SD rats (two-sample unpaired *t* test, *P* = 0.003), suggesting that FH microbiome transplantation could induce IL-17A accumulation in FH rat hippocampus while SD microbiota could not. All the above results indicated that the hippocampal cytokine metabolism should be associated with the mental disorder, and SD fecal microbiota transplantation could ameliorate the dysfunction of hippocampal cytokine metabolism in FH recipients.

10.1128/msystems.00218-22.1 FIG S1 Boxplot showing the concentration of inflammatory cytokines in serum tissues (left panel) and hippocampal samples (right panel). The asterisks indicate as follows: ns, adjusted *P* ≥ 0.05; *, adjusted *P* < 0.05; **, adjusted *P* < 0.01; ***, adjusted *P* < 0.001; ****, adjusted *P* < 0.0001 (Wilcoxon test for all serum cytokines and hippocampal IL-6 and TNF-α; Student’s t-test for hippocampal IL-2, IL-4, IL-10, IL-1β, IL-17A and IFN-γ). Download FIG S1, EPS file, 2.7 MB. Copyright © 2022 Hu et al. 2022 Hu et al. https://creativecommons.org/licenses/by/4.0/ This content is distributed under the terms of the Creative Commons Attribution 4.0 International license.

### FMT-mediated changes in fecal microbial taxonomy.

**FIG 2:**
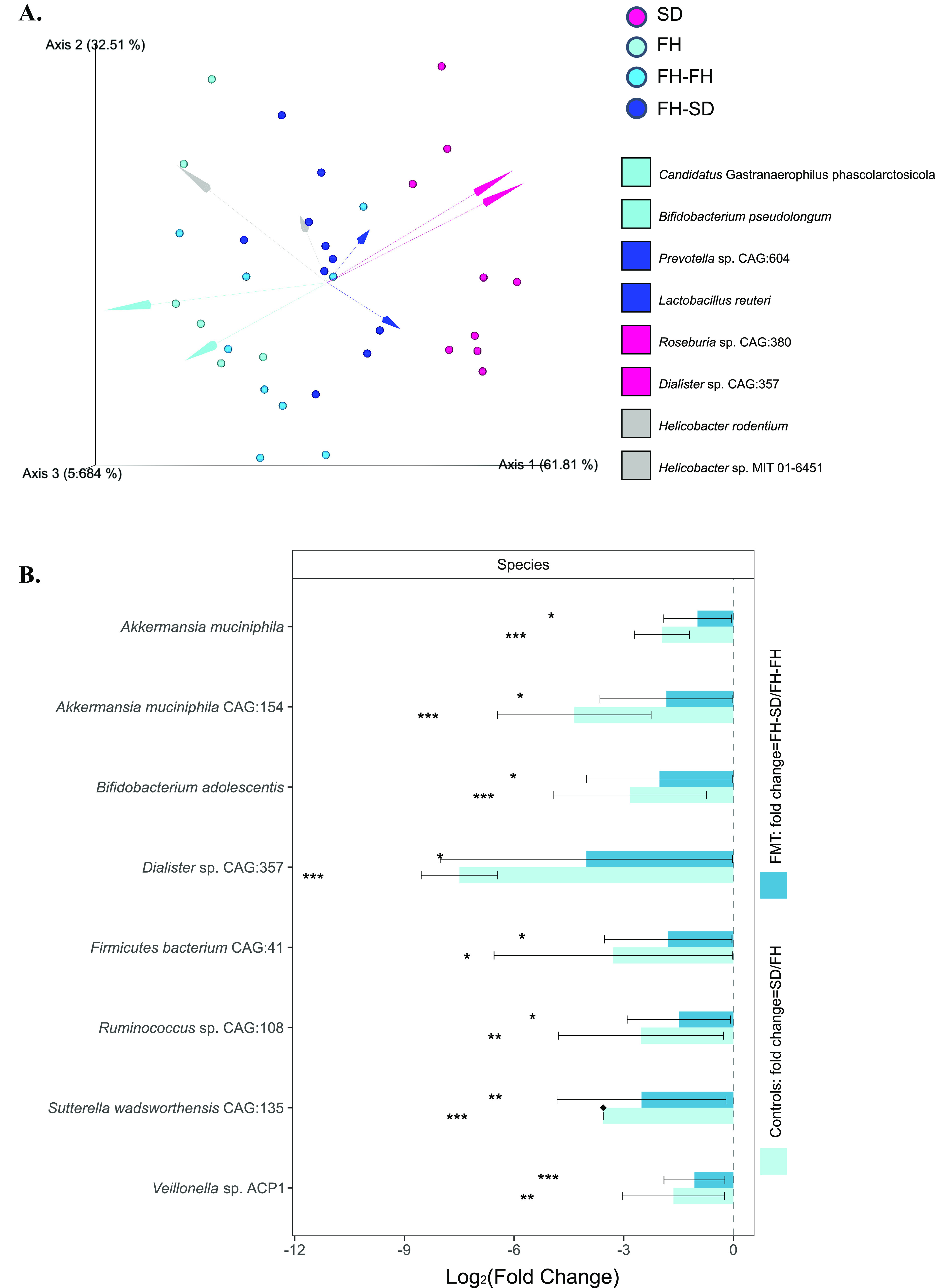
Quantitative analysis of gut microbial diversity and proportions. (A) DEICODE (robust Aitchison PCA) generated biplot. Data points represent individual rats and are colored by group. Taxa driving the ordination space are exemplified by the vectors, labeled with the lowest common ancestor. (B) ANCOM-BC model-derived pairwise differential proportion analysis stratified by control and FMT groups where the data are represented by effect size (log_2_ change) and 95% confidence interval bars (two-sided; Bonferroni adjusted). All effect sizes with adjusted *P < *0.05 are indicated: *, *P* < 0.05; **, *P* < 0.01; ***, *P* < 0.001.

To determine if the gut microbiota in FH-SD rats had been altered, species-level beta-diversity based on 16S rRNA amplicon sequencing data were analyzed using DEICODE. As shown in Fig. 2A, FH and FH-FH rats had significantly different beta diversity compared to FH-SD and SD rats (permutational analyses of multivariate dispersions [PERMDISP]: *F* = 0.230, *P* = 0.818, *n* = 999 permutations; permutational analysis of variance [PERMANOVA]: *F* = 8.689, *P* = 0.001, *n* = 999 permutations). The SD microbiota was significantly differentiated from the FH microbiota by the proportion of *Roseburia* sp. CAG 380 and *Dialister* sp. CAG: 357. Likewise, the FH microbiota was characterized by increased proportions of Bifidobacterium pseudolongum and Candidatus Gastranaerophilus phascolarctosicola. The log ratio of DEICODE-feature loadings of these four species were employed further to examine the proportion of SD:FH-associated species. A significantly greater log ratio of *Roseburia* sp. CAG 380 and *Dialister* sp. CAG: 357 (in the numerator) to Bifidobacterium pseudolongum and Candidatus Gastranaerophilus phascolarctosicola (in the denominator) between FH and SD rats, as well as between FH-FH and FH-SD rats, were observed (Wilcoxon test, *P* < 0.05; Fig. S2). This suggested a successful transfer of the SD gut microbiota to the FH recipients. To further identify differentially proportional taxa and account for the compositional data, Analysis of Compositions of Microbiomes with Bias Correction (ANCOM-BC) was applied. As shown in Fig. 2B, there were eight species with significantly different proportions both between the two control groups (i.e., SD versus FH) and between the two FMT-processed groups (i.e., FH-FH versus FH-SD) (effect sizes with Bonferroni, adjusted *P < *0.05). These differentially proportional species were Akkermansia muciniphila, Akkermanisia muciniphila CAG:154, Bifidobacterium adolescentis, *Dialister* sp. CAG357, Firmicutes bacterium CAG:41, *Ruminococcus* sp. CAG:108, Sutterella wadsworthensis CAG:135, *Veillonella* sp. ACP1 and their proportions were significantly lower in SD and FH-SD rats than in FH and FH-FH rats. Interestingly, *Dialister* sp. CAG357 was the sole pairwise differentially proportional species that could determine the species-level beta-diversity differentiation between SD and FH-SD gut microbiota and the FH and FH-FH gut microbiota (Fig. 2A). Therefore, it was considered that the SD fecal microbiota transplantation was successful in shifting the microbiota of the recipient FH rats toward the SD-characteristic microbiota, and the significant decrease of *Dialister* sp. CAG357 might play a key role in the gut microbiota reassembly in FH-SD rats.

10.1128/msystems.00218-22.2 FIG S2 Box plot illustrating the log-ratios of SD:FH-associated species in experimental groups, where the asterisks indicate as follows: ns, *P* ≥ 0.05; *, *P* < 0.05; **, *P* < 0.01; ***, *P* < 0.001; ****, *P* < 0.0001 (Wilcoxon test). Download FIG S2, EPS file, 0.4 MB. Copyright © 2022 Hu et al. 2022 Hu et al. https://creativecommons.org/licenses/by/4.0/ This content is distributed under the terms of the Creative Commons Attribution 4.0 International license.

### FMT-mediated changes in fecal microbial functional potential.

**FIG 3:**
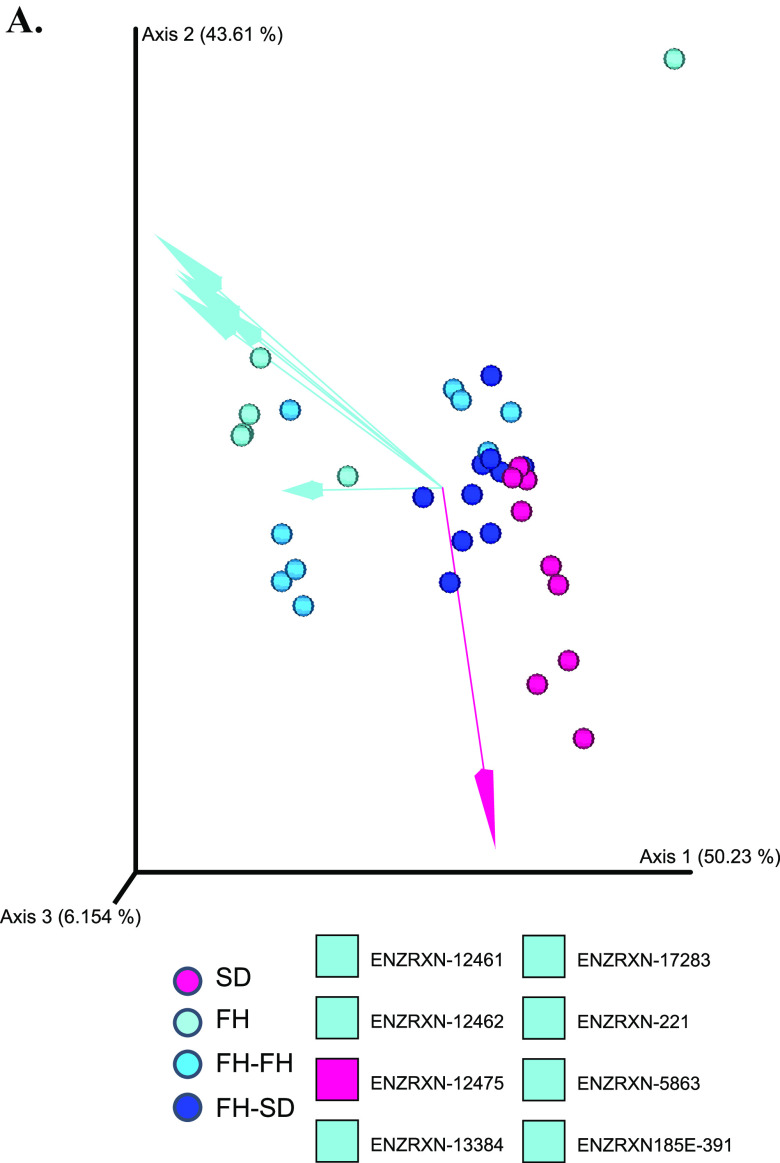

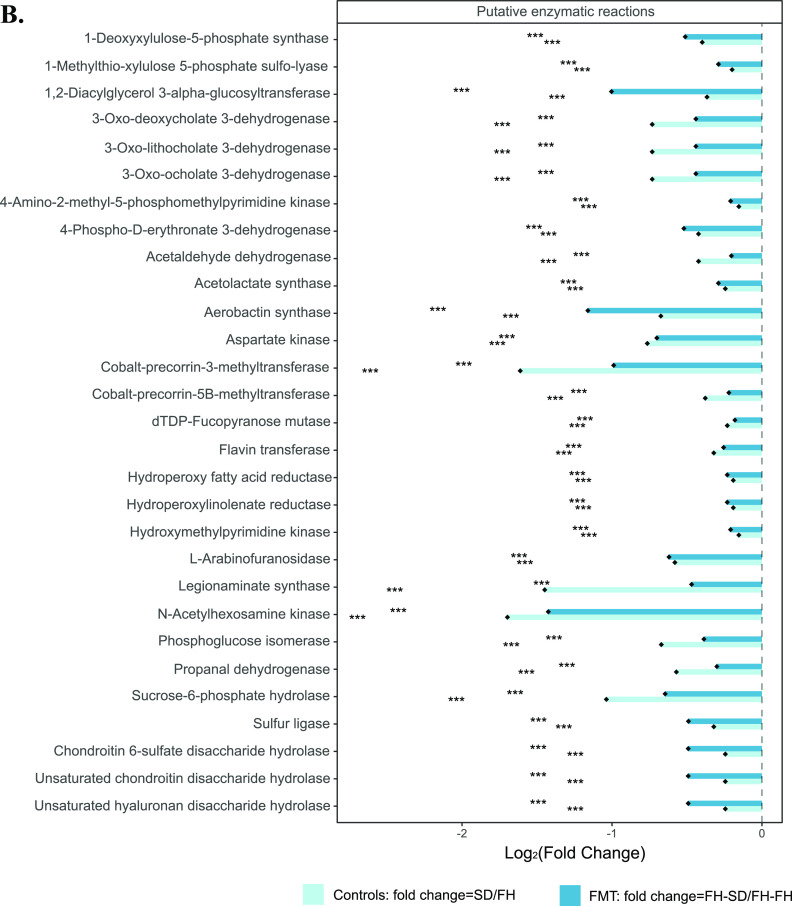
Quantitative analysis of gut functional diversity and abundance in terms of enzymatic reactions. (A) DEICODE (robust Aitchison PCA) generated a biplot of enzyme reactions. Data points represent individual rats and are colored by group, and arrows represent enzymatic genes. ENZRXN-12461, citrate hydro-lyase; ENZRXN-12462m d-threo-isocitrate hydro-lyase; ENZRXN-12475, 1-deoxy-d-xylulose 5-phosphate reductoisomerase; ENZRXN-13384, sucrose phosphorylase; ENZRXN-17263, acetolactate synthase; ENZRXN-221, butyryl-CoA dehydrogenase; ENZRXN-5863, isoamylase; ENZRXN-185E-391, phosphoglucomutase. (B) ANCOM-BC model-derived pairwise differential proportion analysis on enzymatic genes stratified by control and FMT groups, where the data are represented by effect size (log_2_ change) and 95% confidence interval bars (two-sided; Bonferroni adjusted). Diamonds on top of some bars indicate structural zeros. All effect sizes with adjusted *P < *0.05 are indicated as follows: *, *P* < 0.05; **, *P* < 0.01; ***, *P* < 0.001.

To determine if the genetic functional potential of the recipient FH rat microbiome was altered by the donor SD rat microbiome upon FMT, MetaCyc database-mapped enzymatic reactions and pathways for the metagenomic data of the four groups were analyzed using DEICODE beta-diversity. As shown in Fig. 3A, FH and FH-FH rats had significantly different beta-diversities compared to FH-SD and SD rats in terms of enzymatic reactions (PERMDISP: *F* = 1.420, *P =* 0.179, *n* = 999 permutations; PERMANOVA: *F* = 7.155, *P =* 0.001, *n* = 999 permutations). It was found that the group of SD and FH-SD microbiomes was characterized by the gene encoding 1-deoxy-d-xylulose 5-phosphate reductoisomerase, while the clustering of FH and FH-FH microbiomes was characterized by genes encoding citrate hydro-lyase, d-threo-isocitrate hydro-lyase, sucrose phosphorylase, acetolactate synthase, butyryl-CoA dehydrogenase, isoamylase, and phosphoglucomutase. Similar to the taxonomic beta-diversity analysis, the functional beta-diversity in FH-SD rats was more similar to SD rats than to FH and FH-FH rats (Fig. S3A). The metabolic pathways that defined the SD and FH-SD microbiomes were quinate degradation I and II, gallate biosynthesis, urea cycle, and carbamoyl-phosphate synthesis, while the pathways that characterized the FH and FH-FH microbiomes were l-citrulline biosynthesis, l-citrulline degradation, and l-proline biosynthesis II (from arginine). Quinate was one of several aromatic compounds that can be metabolized by microorganisms to the central intermediate protocatechuate and then be further metabolized via the β-ketoadipate pathway to acetyl-CoA and succinyl-CoA. According to a Spearman’s rank correlation analysis between the robust-Aitchison principle component analysis (RPCA) generated distance-ordination matrices of functional genes at the reaction level (along the X-axis) and pathway level (along the y-axis), it was demonstrated that there was a significant association between the pathways and enzyme reactions with ρ = 0.8741 (over 999 permutations, *P* = 0.001; Fig. S3B). By applying ANCOM-BC, the pairwise differentially proportional enzyme-encoding genes and pathways in both the control groups (i.e., SD versus FH) and FMT-processed groups (i.e., FH-FH versus FH-SD) were identified. Nine metabolic pathways were pairwise differentially proportional, including glycolipid biosynthesis, chondroitin sulfate degradation, cytidine monophosphate (CMP)-legionaminate biosynthesis, dermatan sulfate degradation, pyruvate fermentation-acetoin I, pyruvate fermentation-acetoin, starch degradation II, and zwittermicin A biosynthesis (Fig. S3C). As shown in Fig. 3B, there were 29 pairwise differentially abundant enzyme genes, all of which were significantly less abundant in SD or FH-SD microbiomes than those in FH or FH-FH microbiomes, respectively (effect sizes with Bonferroni, adjusted *P < *0.05). Most of the pairwise differentially abundant genes were associated with carbon metabolism. Among them, the gene encoding acetolactate synthase, which could catalyze the conversion between pyruvate and 2-acetolactate and was involved in valine and isoleucine biosynthesis and then pantothenate and CoA biosynthesis, was the only one associated with the beta-diversity differentiation between SD/FH-SD and FH/FH-FH (Fig. 3A). Hence, SD-FMT significantly changed the microbiome profile of FH rats, resulting in a significant reduction in the proportion of genes encoding acetolactate synthase.

10.1128/msystems.00218-22.3 FIG S3 Quantitative analysis of gut functional diversity and abundance in terms of MetaCyc pathways. (A) DEICODE (robust Aitchison PCA) generated a biplot of MetaCyc pathways. CARBPSYN-RXN, carbamoyl phosphate synthesis; CITRULBIO-PWY, l-citrulline biosynthesis; CITRULLINE-DEG-PWY, l-citrulline degradation; PWY-4981, l-proline biosynthesis II (from arginine); PWY-4984, urea cycle; PWY-6416, quinate degradation II; PWY-6707, gallate biosynthesis; and QUINATEDEG-PWY, quinate degradation. (B) Mantel scatter plot between the MetaCyc-mapped enzymatic reactions and pathway assignments of robust-PCA-based distance matrices. (C) ANCOM-BC model-derived pairwise differential proportion analysis on metabolic pathways stratified by control and FMT groups, where the data are represented by effect size (log_2_ change) and 95% confidence interval bars (two-sided; Bonferroni adjusted). Diamonds on top of some bars indicate structural zeros. All effect sizes with adjusted *P* < 0.05 are indicated as follows: *, *P* < 0.05; **, *P* < 0.01; ***, *P* < 0.001. Download FIG S3, PDF file, 2.1 MB. Copyright © 2022 Hu et al. 2022 Hu et al. https://creativecommons.org/licenses/by/4.0/ This content is distributed under the terms of the Creative Commons Attribution 4.0 International license.

### Microbe-metabolite co-occurrences among the group-associated features.

**FIG 4:**
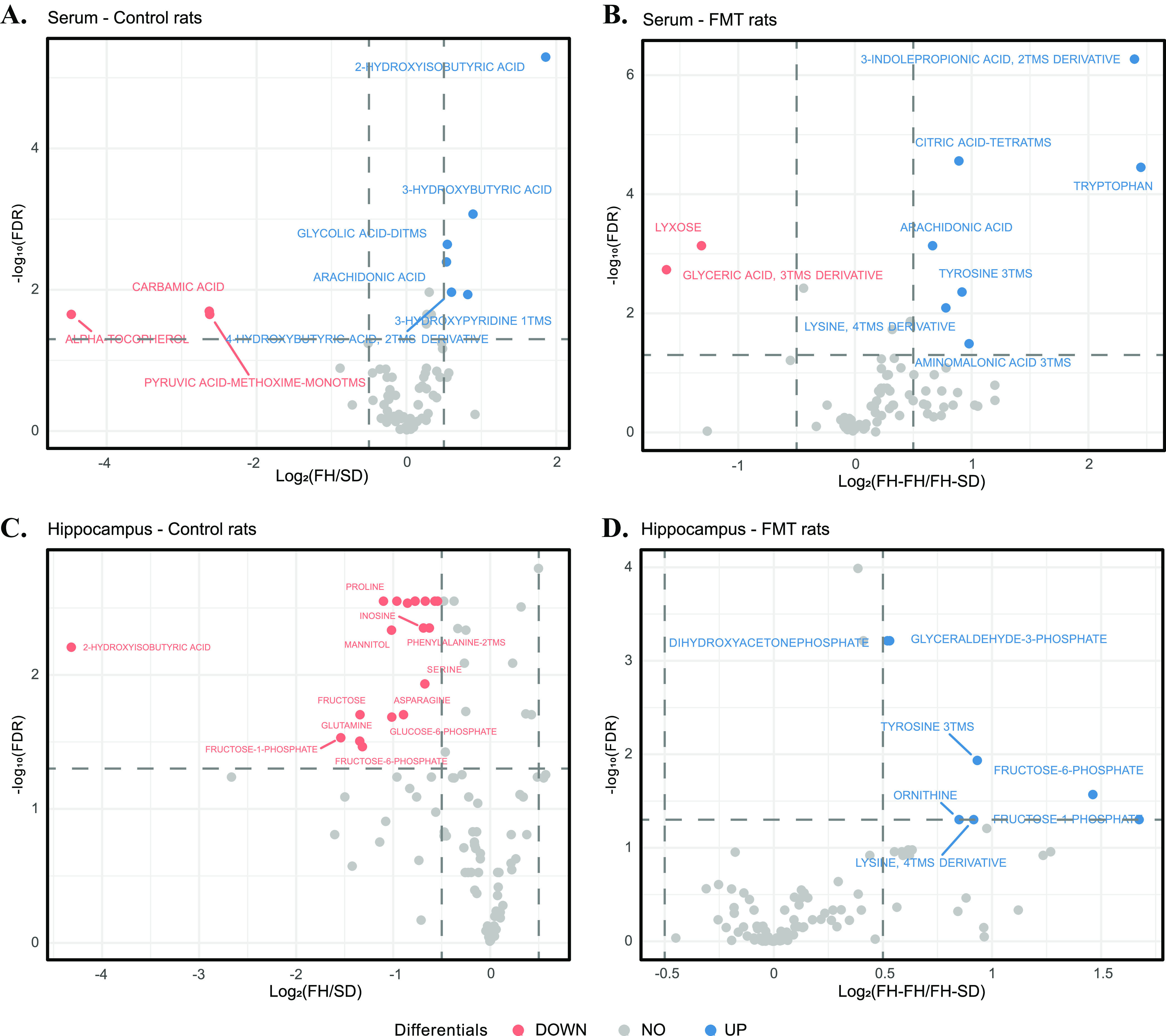
Volcano plots showing the differential serum metabolites in between FH and SD rats (A) and between FH-FH and FH-SD rats (B), and the volcano plots showing the differential hippocampal metabolites between FH and SD rats (C) and between FH-FH and FH-SD rats (D). Log_2_ change > 0.5, FDR value < 0.05 (in blue): significantly higher in FH rats than that in SD rats (control rats) or FH-FH rats than that in FH-SD rats (FMT rats), respectively; Log_2_ change < 0.5, FDR value < 0.05 (in red): significantly reduced in FH rats than that in SD rats (control rats) or FH-FH rats than that in FH-SD rats (FMT rats), respectively.

Serum and hippocampal metabolomics were performed for the four groups of rats. Comparative metabolomics analysis demonstrated that serum metabolomic profiles were relatively conserved between all four rat groups (Fig. 4A and B), but hippocampal metabolite profiles were considerably different between control rats and FMT-processed rats (Fig. 4C and D). Similarly, DEICODE-generated biplots of serum metabolomics (Fig. S4A) showed differentiation of SD and FH-SD rats to FH and FH-FH rats (PERMDISP: *F* = 0.929, *P =* 0.382, *n* = 999 permutations and PERMANOVA: *F* = 4.907, *P =* 0.001, *n* = 999 permutations), while hippocampus metabolic diversity of SD rats showed distinction with those of the FMT-processed and control FH rats (PERMDISP: *F* = 1.113, *P =* 0.276, *n* = 999 permutations; PERMANOVA: *F* = 12.617, *P =* 0.001, *n* = 999 permutations) even though the hippocampal metabolomes of FH-SD rats had the trend toward those of SD rats (Fig. S4B). These results indicated that the transplantation of SD gut microbiota to FH rats had a greater effect on the recipients’ serum metabolism than hippocampal metabolism. Among the significantly differentially abundant metabolites in control and FMT-processed rats, arachidonic acid (C20:4) in serum was the sole metabolite that was significantly pairwise differentially abundant in FH and FH-FH rats, compared to SD and FH-SD rats. The abundance of serum C20:4 in FH-SD rats was significantly lower than that in FH-FH rats (Wilcoxon test, *P = *0.0007) and was similar to that in the SD donor (*P = *0.243). This indicated that the transplantation of SD gut microbiota reduced the serum concentration of C20:4 in FH rats.

10.1128/msystems.00218-22.4 FIG S4 DEICODE-generated biplots of metabolomics in serum (A) and hippocampus (B). The data points represented individual rats and were colored by group. Metabolites driving the ordination space were exemplified by the vectors. Download FIG S4, PDF file, 2.9 MB. Copyright © 2022 Hu et al. 2022 Hu et al. https://creativecommons.org/licenses/by/4.0/ This content is distributed under the terms of the Creative Commons Attribution 4.0 International license.

Meanwhile, by doing the correlational analyses on the serum and hippocampal metabolomes, respectively, the associative nature of metabolites among themselves was identified. Interestingly, it was found that several carbon-rich fatty acids (i.e., palmitic acid (C16:0), heptadecanoic acid (C17:0), stearic acid (C18:0), and linoleic acid (C18:2)) and nitrogen-rich metabolites (e.g., 2-aminoethanol, glucopyranose, glycine, and uracil) had strongly inverse relationships in rat serum (Fig. S5A), while numerous nitrogen-rich metabolites (e.g., serine, ornithine, asparagine, and glutamine) had highly negative relationships with several brain osmolyte compounds or their precursors (i.e., N-acetylaspartic acid [NAA], inositol, and pyroglutamic acid) in rat hippocampus (Fig. S5B). Considering that the level of C20:4, a derivative of C16:0, C17:0, C18:0, and C18:2 in lipid metabolism, was pairwise significantly reduced in the serum of SD and FH-SD rats as shown in Fig. 4A and B, the contents of nitrogen-rich metabolites in serum of SD and FH-SD rats were expected to be higher in comparison with those in FH and FH-FH rats regardless of the significance, which would result in the increase of amino acids and decrease of osmolytes in the hippocampus through the blood-brain barrier (BBB). This inference was supported by the hippocampal metabolomes of the four groups of rats, where the log ratio of serine to NAA was significantly higher in FH-SD and SD rats than that in FH rats (Wilcoxon test, *P* < 0.05; Fig. S6).

10.1128/msystems.00218-22.5 FIG S5 Correlation heatmaps of serum metabolites (A) and hippocampal metabolites (B). Blue color indicates a positively significant association, red color indicates a negatively significant association and white-colored cells are the insignificant correlations (*P* ≥ 0.05). Download FIG S5, JPG file, 2.7 MB. Copyright © 2022 Hu et al. 2022 Hu et al. https://creativecommons.org/licenses/by/4.0/ This content is distributed under the terms of the Creative Commons Attribution 4.0 International license.

10.1128/msystems.00218-22.6 FIG S6 Boxplot showing the log-ratio of serine to N-acteyl-aspartate in hippocampal metabolomics. The asterisks indicate as follows: ns, *P* ≥ 0.05; *, *P* < 0.05; **, *P* < 0.01; ***, *P* < 0.001; ****, *P* < 0.0001 (Wilcoxon test). Download FIG S6, EPS file, 0.8 MB. Copyright © 2022 Hu et al. 2022 Hu et al. https://creativecommons.org/licenses/by/4.0/ This content is distributed under the terms of the Creative Commons Attribution 4.0 International license.

To examine the co-occurrences between metabolomes in host tissues and specific bacterial species in gut microbiota, we employed MMvec, which uses neural networks to infer the nature of interactions across omics data sets. The heatmap reflecting the conditional probabilities between the serum metabolomes and DEICODE-associated and ANCOM-BC-associated bacteria taxa along principal component 1 (PC1) (Fig. S7A) suggested a strong likelihood of co-occurrence for all pairings with positive and higher conditional probabilities. The model showed a higher predictive accuracy of Q^2^ = 0.35 than the absolute null or baseline model (where no formula was used) on the cross-validation samples (Fig. S7B). Nevertheless, the hippocampal metabolomes did not show a good prediction of co-occurrences with the DEICODE-associated and ANCOM-BC-associated microbes (Q^2^ ≈ 0), hence no visualized data were shown here. This confirmed the expectation that the intestinal microbiome more strongly influenced serum metabolites than hippocampal metabolites.

10.1128/msystems.00218-22.7 FIG S7 Microbe-metabolite co-occurrences amongst the group-associated features clustered heatmap showing the log conditional probabilities between microbial species or functions and metabolites present in the serum (A) along with convergence summary of the models (B). Download FIG S7, PDF file, 0.9 MB. Copyright © 2022 Hu et al. 2022 Hu et al. https://creativecommons.org/licenses/by/4.0/ This content is distributed under the terms of the Creative Commons Attribution 4.0 International license.

## DISCUSSION

**FIG 5:**
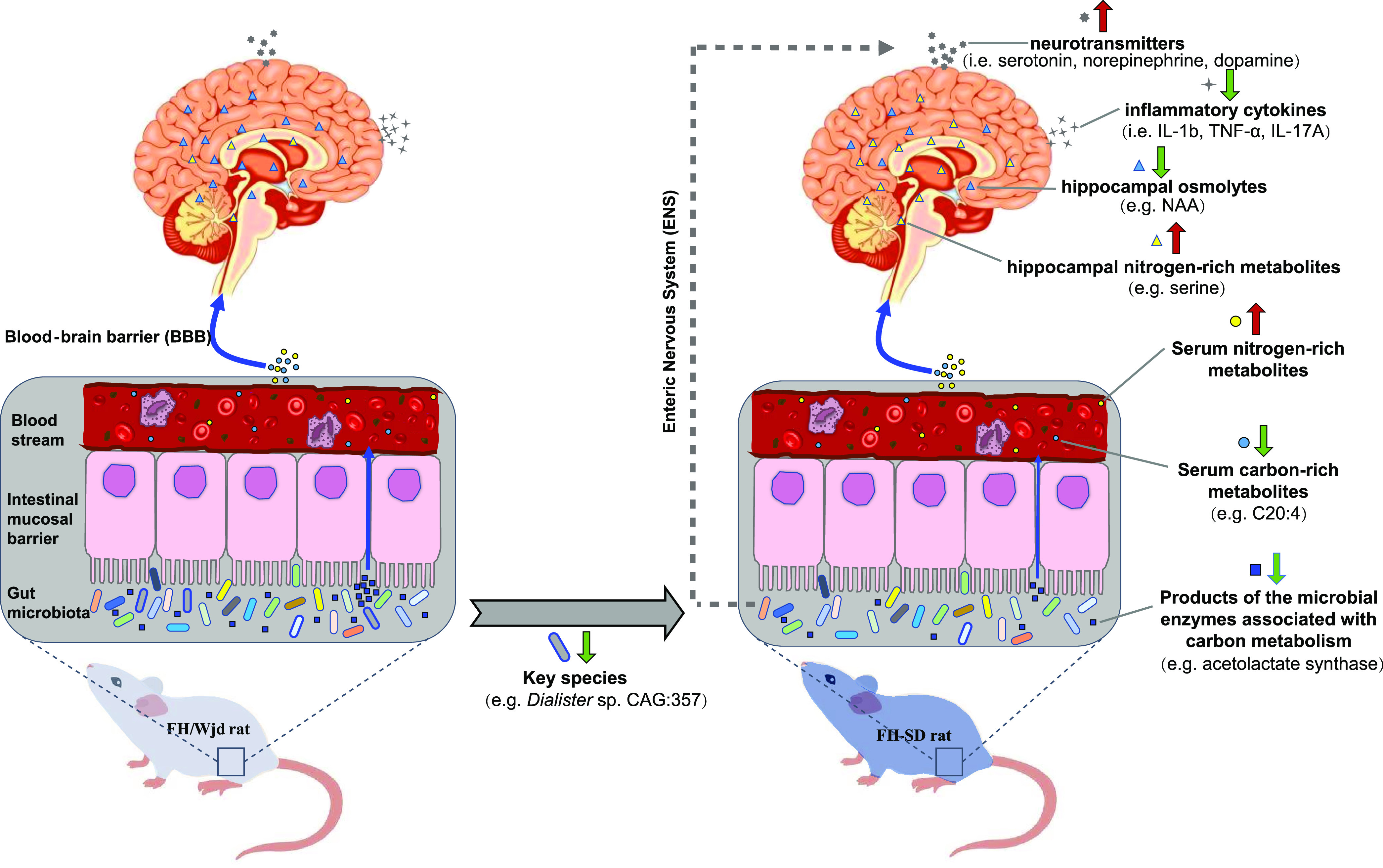
A predicted mechanistic overview for the potential therapeutic impact of FMT resulting in a reduction in depression-like behaviors in recipient hosts. The cobalt blue arrows represent the effect of gut microbiota on neurogenesis and hippocampal inflammation through the blood-brain barrier (BBB); the dotted gray arrow represents the potential effects of the gut microbiota on neurogenesis directly through the enteric nervous system (ENS). Compounds in different tissues are represented by different geometric shapes. The thick red and green arrows represent the increasing and decreasing levels of the responding objects, respectively. C20:4 = arachidonic acid; NAA = N-acetylaspartic acid.

Previous investigations of the gut microbiome and depression have focused on a cross-sectional analysis of healthy and depressed subjects (32), or between germfree and depression-associated microbiome colonized subjects (12). Our study represents the first to determine if it is possible to alter a depression-like phenotype with fecal microbiota transplants from ‘healthy’ animals by using the male FH and SD rats of the same age. It was found that FMT from FH donors to FH rats aggravated the recipients’ depressive symptoms, but FMT from SD donors to FH rats alleviated the deterioration (Fig. 1A to C). Additionally, monoamine neurotransmitter concentrations were more or less increased in the hippocampus (Fig. 1E and F), and three hippocampal immune cytokines (i.e., IL-1β, TNF-α, and IL-17A) were significantly reset (Fig. S1). A recently similar study in mice demonstrated that gut microbiota from inflammasome NLRP3-deficient mice, whose production of proinflammatory cytokines was limited, ameliorated depressive symptoms in the recipient wild-type mice, but the key gut microbes and their detailed therapeutic mechanism were not investigated (33). Multiomics associated analyses on the four groups of rats in the study suggest that FMT directs gut microbiome modulation, and results in systematic metabolic modulation in the recipients through the intestinal mucosal barrier, ENS, and the BBB. We represent this hypothesis in Fig. 5, where the gut microbial community compositions of the four groups of rats (Control FH, Control SD, FH-FH, and FH-SD) were characterized. It was found that transplantation of SD fecal microbiota successfully shifted gut microbiomes of the recipient FH rats toward the SD-characteristic microbiome, with a significant reduction in the proportion of several species such as *Dialister* sp. CAG:357 (Fig. 2) and many carbon metabolism-related enzyme-encoding genes such as acetolactate synthase (Fig. 3). With the associated analyses on serum and hippocampus metabolomics profiles (Fig. 4 and Fig. S4 to S7), it was inferred that the significant shift in the gut microbiome of the FH recipients resulted in repression of their carbon metabolism, leading to a reduction in the abundance of carbon-rich metabolites (e.g., C20:4) and an increase in nitrogen-rich metabolites. The increase in nitrogen-rich metabolites in serum may translate to the brain through the BBB, increasing amino acids synthesis in the hippocampus (e.g., serine) and decreasing the concentration of brain osmolytes (e.g., NAA). The level of hippocampal NAA has been noted in several human brain disorders (34, 35). Even though its functional roles remain unclear, it is believed to be involved in neuromodulation, which is supported by one interpretation of the results presented here. Alternatively, because depression-biomarkers in the serum of FH-SD rats were not significantly different from that of FH and FH-FH rats (Fig. 1D and Fig. S1), and the hippocampal metabolites showed no significant co-occurrence with differentially proportional gut microbes. Transplanted microbes could ameliorate depressive symptoms in recipients by direct neuromodulation through the ENS, potentially via the vagus or other efferent nerves.

The ENS, an intricate network consisting of more than 500 million neurons and glia within the bowel wall, does not only control bowel motility, epithelial secretion, and intestinal immunity (36) but also communicates with the CNS through the vagus and other efferent nerves. Thus, direct stimulation or disruption of the ENS may result in CNS disturbance. Such a mechanism has been proposed for conditions such as Parkinson’s disease (37) and autism spectrum disorder (38, 39). The gut microbiome has been associated with direct ENS stimulation and interaction, such as during ENS development in infants (40). De Vadder et al. (41) found that the germ-free adult mice had immature ENS, and the situation was rescued by gut microbiota from conventionally raised mice, which induced neuronal and mucosal serotonin production and the proliferation of intestine neuronal progenitors. Nevertheless, because the causal relationship between glial bioenergetics and depressive symptoms is not clear yet (42), the detailed mechanisms underpinning such microbiome effects are still hypothetical (43).

In our study, a decreased proportion of *Dialister* sp. CAG:357 was considered to be associated with the prevention of depression development in FH-SD rats. *Dialister* spp. is common obligate anaerobes in the human microbiome (44, 45), but in conflict with our results, Valles-Colomer et al. (46) found that *Dialister* spp. was consistently depleted in humans with depression. However, this difference could result from differences in the hosts (rat versus human) or suggests that *Dialister* is potentially not involved at all in the observed behavioral responses.

### Conclusions.

In conclusion, we demonstrated that gut microbiota transplantation from ‘healthy’ SD rats to FH rats could suppress the aggravation of depression symptoms in the recipients through the gut microbe-induced modulation of host immune and metabolic activity. This study took a first step toward understanding the potential of FMT as a therapy for depression.

## MATERIALS AND METHODS

### Animal models.

Thirty male Fawn-hooded (FH) rats and 10 healthy male Sprague–Dawley (SD) rats at the age of 3 weeks old and of nonspecific pathogen-free (SPF) grade were purchased from the Department of Laboratory Animal Science at Beijing University Health Science Center (PUHSC). All the rats (*n* = 40) were prehoused in cages (i.e., *n* = 5 rats per cage) for 3 days in the animal experimental room at PUHSC under SPF conditions for acclimatization. They were housed at 22 ± 1°C with a humidity of 70% on 12 h-day:12 h-night cycles (lights on 7:00 to 19:00) and were given a standard laboratory chow diet and water *ad libitum*.

### Fecal microbiota transplantation.

From a total of 30 FH rats and 10 SD rats, 10 FH rats and 10 SD rats were used as donors for the fecal microbiota transplantation (FMT), respectively. Fecal pellet samples were collected a day before the FMT experiment and processed as described by Zheng et al. (12). Briefly, the fecal samples were mixed with sterile phosphate-buffered saline (PBS, pH 7.0) at a weight-to-volume ratio of 1:6 under anaerobic conditions, and then centrifuged at 800 × *g* for 2 min to discard the fecal debris. The microbiota in the supernatants was stored overnight at 4°C for the FMT on the following day. The remaining 20 FH rats were divided into two equal-sized groups based on equivalent average body weight. The fecal extracts from the FH and SD donor rats were administered to the first group named ‘FH-FH’ and the second group named ‘FH-SD’ rats, respectively. Each inoculation volume was 0.5 mL performed at a total of eight times around a single-day interval. In parallel to these experimental groups, an additional control group of 10 FH rats and 10 SD rats were treated with PBS under the same incubation conditions.

### Neurobehavioral tests.

Animal model rats were examined for their neurobehaviors using the following commonly conducted behavioral tests: forced swim test (FST), open-field test (OFT), and sucrose preference test (SPT). Each test was performed post-FMT in the animal experimental room at PUHSC on three separate days with prior acclimation for 1 h. The details of the testing operations are as follows:

### FST.

The experiment was carried out according to Ge et al. (47). The rats were placed individually in a PVC-made translucent cylinder (50 cm in height × 20 cm in diameter) filled with 30 cm water (22.5 ± 0.5°C), in which the rats could not support themselves touching the bottom with their bodies. The testing paradigm included two sections: an initial 15 min pretest for acclimation, and then a 5-min test 24 h later. A video camera was held near the cylinder to record the duration of the rat's immobile state in the second section. Immobility was defined such that all motions of the rat limbs stroke was absent except for movements required to keep the rat’s head above water.

### OFT.

All the rats were individually tested in an open-field apparatus consisting of a gray square base (100 × 100 cm^2^) with gray walls (40 cm in height), made up of polyvinyl chloride (PVC). Each rat was gently placed in the center of the chamber, and its spontaneous activity was recorded for 10 min using the video-computerized tracking system, which was set at 100 cm above the chamber. For recording purposes, the base area was equally arranged into 5 × 5 squares, and the value of the total square amounts in which the experimental rat set foot during the 10-min testing was used as an index of locomotor activity, while the proportion of central squares (inner 36% of the base area) to the total squares in which the experimental rat set foot during the 10-min testing was construed as an index of anxiety-like behavior. The chamber was cleaned up before the next rat was placed in.

### SPT.

Two no-drip pet water feeding bottles containing 1.0% (wt/vol) sucrose were hung on different sides of the experimental cage. Before conducting the experiment, rats were mono housed in the experimental cage with the provision of two water bottles for 48 h to overcome neophobia, followed by exposure to a 6-h period of water and food deprivation. Next, the solutions in the two bottles were each replaced with 80 mL of 1% sucrose and plain water, respectively. Each experimental rat was provided with open access to the two bottles hanging on each side of the cage for 30 min. Later, the sucrose and plain water bottles were switched, and the open access was provided for another 30 min, to eliminate side-preference artifacts. Each of the bottles was weighed before and after use. The sucrose preference index of each rat was computed according to the following formula I_SP_ = ×100%, where I_SP_ was the sucrose preference index of each rat, ΔM_s_ was the weight difference of the sucrose bottle before and after SPT, and ΔM_w_ was the weight difference of the water bottle before and after SPT.

### Extraction of neurotransmitters, immune cytokines, and metabolites from the hippocampal tissues and serum.

One-week post-FMT, the control, and experimental rats were sacrificed to obtain the hippocampal and serum samples. The hippocampal tissue from each rat was divided into two aliquots, one for sample preparation and quantification of neurotransmitters and immune cytokines, and the other one for sample preparation and quantification of untargeted metabolomics.

Sample preparation for neurotransmitters and immune cytokines was processed as follows: the hippocampal tissue was weighed and homogenized in the lysis buffer (composition consists of 10 mM HCl, 1 mM EDTA, 4 mM Na_2_S_2_O_5_) to a ratio of 6 mL lysis buffer to 1 mg tissue sample. The mixed homogenate was then centrifuged at 18,000 × *g* and 4°C for 10 min. The supernatant was filtered using a 0.2 μm Millipore filter (Millipore, MA, United States), and stored at −80°C until further use.

Sample preparation for metabolomics was processed as follows: The hippocampal tissue was desalinated using methanol, i.e., 20 mg of the hippocampus tissue was mixed with 800 mL methanol-water (4:1 vol/vol) solution containing 5 μg/mL myristic acid-1,2-^13^C_2_, which was used as the internal standard for the metabolomics. This mixture was further milled using the rotor beater mill (Retsch, Haan, Germany) three times, incubated at 4°C for 60 min, and centrifuged at 20,000 × *g* and 4°C for 10 min. The supernatant was filtered using a 0.2 μm Millipore filter and stored at −80°C until further derivatization and analysis. One hundred twenty microliters of the desalinated hippocampal extract were added to an MS-certified glass vial equipped with a 200 μL insert (Freeze-Dryer, Boyikang, Beijing, China) for 4 h of lyophilization. This lyophilized material was derivatized using a method analogous to Moros et al. (48), where the lyophilized samples were mixed with 30 μL methoxyamine (10 mg/mL) in pyridine for 16 h at room temperature for the methoxyamination followed by the trimethylsilylation with 30 μL of N-methyl-N-(trimethylsilyl) trifluoroacetamide (MSTFA) with 1% trimethylchlorosilane (TMCS). Subsequently, 30 μL of methyl myristate in *n*-pentadecane was added as an injection external standard.

For serum metabolomics, 50 μg of fresh serum sample was extracted with 200 μL of a cold methanol-water solution containing the internal standard myristic acid-1,2-^13^C_2_. Later, the mixture was centrifuged, lyophilized, and derivatized in a similar method to that used for hippocampal samples (as described above).

### ELISA-based quantification of neurotransmitters in the hippocampus and serum.

Absolute quantification of serotonin in the hippocampal-tissue- and serum extracts was determined using the serotonin-specific enzyme-linked immunosorbent assay (ELISA) kit DEE5900 and DEE8900 (Demeditec Diagnostics GmbH, Kiel, Germany), respectively. The concentrations of noradrenaline in the hippocampal-tissue-extracts were determined with the noradrenaline-sensitive ELISA kit BCU39-K01 (Eagle Biosciences Inc., Amherst, NH, United States). All the samples were run in three biological replicates on each ELISA microplate.

### Gas chromatograph- mass spectrometer (GC-MS)-based quantification of host and microbial metabolites in the hippocampus and serum.

Metabolite profiling of the derivatized samples was performed on a GCMS-QP2010 (SHIMADZU, Kyoto, Japan). A 0.5 μL of the derivatized sample was injected into an RTx-5MS column (30 m × 0.25 mm × 0.25 μm, Restek Corp., PA, USA) with helium as carrier gas at a constant flow of 1.5 mL/min. The inlet temperature was set to be 250°C. The initial oven temperature was held at 80°C, ramped to 300°C by 20°C per min, and then held at 300°C for 3 min. Electron impact was used as an ionization source with the ionization energy of 70 eV at 200°C. The transfer line temperature was set to be 220°C. Mass spectra were recorded at 50 to 700 *m/z* for 4.5 to 18 min. Metabolites were identified based on the mass spectrum in comparison to the standard NIST library 2.0 (National Institute of Standards and Technology, 2008) and Wiley 9 (Wiley-VCH Verlag GmbH & Co. KgaA, Weinheim, Germany), with a threshold of match > 80 (with a maximum match equal to 100). Relative metabolite abundances were calculated from peak areas (unique mass) of identified metabolites using GCMS LabSolution software and followed by calibration using the peak area of the internal standard (myristic acid-1,2-^13^C_2_) and the external standard (methyl myristate) to minimize the instrumental errors.

### AimPlex multiplex immunoassays-based quantification of cytokines in the hippocampus and serum.

To detect the immune cytokines in rat hippocampus and serum, an AimPlex multiplex immunoassay kit (Beijing Quantobio Biotechnology Co. Ltd., Beijing, China) was used according to the manufacturer’s instructions. This assay combined the techniques in ELISA and high-throughput flow cytometry and could detect several proteins from very small samples quickly. Here, eight inflammatory cytokines were measured, including IL-4, IL-10, TNF-α, IL-1β, IL-2, IL-6, IL-17A, and IFN-γ.

### Nucleic-acid extraction and sequencing.

One-week post-FMT, fecal samples were collected from the control and experimental rats and were processed for 16S rRNA amplicon sequencing and shotgun metagenomic sequencing. DNA extraction for amplicon-based sequencing was processed using the QIAamp Fast DNA Stool Minikit (Qiagen, Duesseldorf, Germany) to extract the microbial genomic DNA. The V3-V4 region of the 16S rRNA gene was PCR-amplified from the DNA samples using the universal primer 338F (5′-ACTCCTACGGGAGGCAGCA-3′) and 806R (5′-GGACTACHVGGGTWTCTAAT-3′) for Illumina HiSeq paired-end sequencing. DNA extraction for shotgun-based sequencing was processed using the NEBNext Ultra™ DNA Library Prep kit (New England BioLabs, MA, USA) to build the sequencing library from a total amount of 1 μg DNA per sample. The generated library was sequenced on the Illumina HiSeq2500 platform.

### Analysis of 16S rRNA sequencing.

Demultiplexed sequencing data were quality filtered by trimming to 150 bases and were denoised using Deblur (49) through Qiita (50) using the default to generate amplicon sequencing variants (ASVs). The deblurred sequence fragments were inserted into the Greengenes Database (v.13_8) phylogenetic tree using SATé-enabled phylogenetic placement (51). The final feature table obtained from Qiita is composed of 37 samples and 7,467 features. A rarefied data table of 5,000 reads per sample was employed for performing downstream data analyses using Qiime2 (52).

### Analysis of shotgun sequencing.

Quality control-filtered paired-end sequencing reads were then concatenated, converted to Fasta format, and processed by the SHOGUN align function (53) and associated Web of Life phylogenetic database (54). SHOGUN-aligned files were then utilized by Woltka (https://github.com/qiyunzhu/woltka) for gOTU table generation and functional pathway characterization on a per-sample basis. This tool maps sequencing reads to microbial genes based on their associated genomic coordinates to compute microbial functional units (e.g., MetaCyc pathways, protein, enzyme, reaction, and pathway information) (55). In doing so, it avoids microbial functional profiling based on the presence or absence of predefined marker genes. The gOTU table was filtered to remove microbial features per sample with less than 0.001% of relative abundance, leaving 1,543 out of 5,842 gOTUs (with the rank-none parameter) and leaving 894 out of 3,325 gOTUs (with the rank-free parameter) found across all samples.

### Statistical analysis and visualization.

To test the normality of the data distribution, the Shapiro-Wilk test was applied. Accordingly, either parametric tests such as the two-sample *t* test and one-way ANOVA or nonparametric tests such as the Wilcoxon test and the Kruskal-Wallis test were used on the pairwise comparison groups and multigroup comparisons, respectively. To check the variances between the groups, Bartlett’s test was applied. The feature abundance table generated from Qiita (taxonomic assignments) and Woltka (functional assignments) was used as input for beta-diversity RPCA (using DEICODE) to calculate between-group beta diversity in QIIME2 (56). The beta diversity significance within and among groups was examined by the QIIME2 diversity plugin with PERMDISP and PERMANOVA tests. The resulting PCoA and the biplots were visualized using the QIIME2 plugin Emperor (57). Distance matrices used for between-group differences were tested using PERMANOVA and permuted t tests in QIIME2. The feature loadings in the biplot axis with the most difference in groups were visualized using Qurro (58). The abundance of the highest- and lowest-ranked features were used to compute log ratios in different rat groups. ANCOM-BC was used to calculate the pairwise differential species (59). To estimate the conditional probability of a metabolite abundance given the presence of a single microbe, a log-transformed conditional probability matrix from each cross-omics feature pair, i.e., metagenomics (based on the species-level metagenomic data) and metabolomics was built using a neural network algorithm MMvec (60). The GC-MS metabolomics data were filtered for central carbon metabolites in the hippocampal tissue and serum extract. A *post hoc* method Benjamini-Hochberg test was applied to control false discovery rate (FDR) during multigroup comparisons and adjusted *P* values were reported wherever applicable.

### Data availability.

The raw SHOTGUN sequencing data were deposited in the National Microbiology Data Center (NMDC) under the accession number NMDC10017888, and the website is as follows: https://nmdc.cn/resource/genomics/sra/detail/NMDC40013589.
