# Erratum for Hu et al., “Effects of ‘Healthy’ Fecal Microbiota Transplantation against the Deterioration of Depression in Fawn-Hooded Rats”

**DOI:** 10.1128/msystems.00953-22

**Published:** 2022-10-18

**Authors:** Bing Hu, Promi Das, Xianglin Lv, Meng Shi, Jiye Aa, Kun Wang, Liping Duan, Jack A. Gilbert, Yong Nie, Xiao-Lei Wu

## ERRATUM

Volume 7, no. 3, e00218-22, 2022, https://doi.org/10.1128/mSystems.00218-22. There are errors in affiliations a, d, f, and g. The corrected affiliations appear below.

^a^College of Engineering, Peking University, Beijing, People’s Republic of China

^d^Peking University Hospital of Stomatology, Beijing, People’s Republic of China

^f^Peking University Third Hospital, Beijing, People’s Republic of China

^g^Institute of Ecology, Peking University, People’s Republic of China

Page 12, Materials and Methods, line 3, “Beijing” should read “Peking.”

Page 15, Acknowledgments, line 2, “Beijing” should read “Peking.”

