# Supplementary figures and images for: Effects of ‘Healthy’ Fecal Microbiota Transplantation against the Deterioration of Depression in Fawn-Hooded Rats
Source: mSystems. 2022 Apr 28;7(3):e00218-22. doi: 10.1128/msystems.00218-22 (PMC9239139; doi:10.1128/msystems.00218-22)

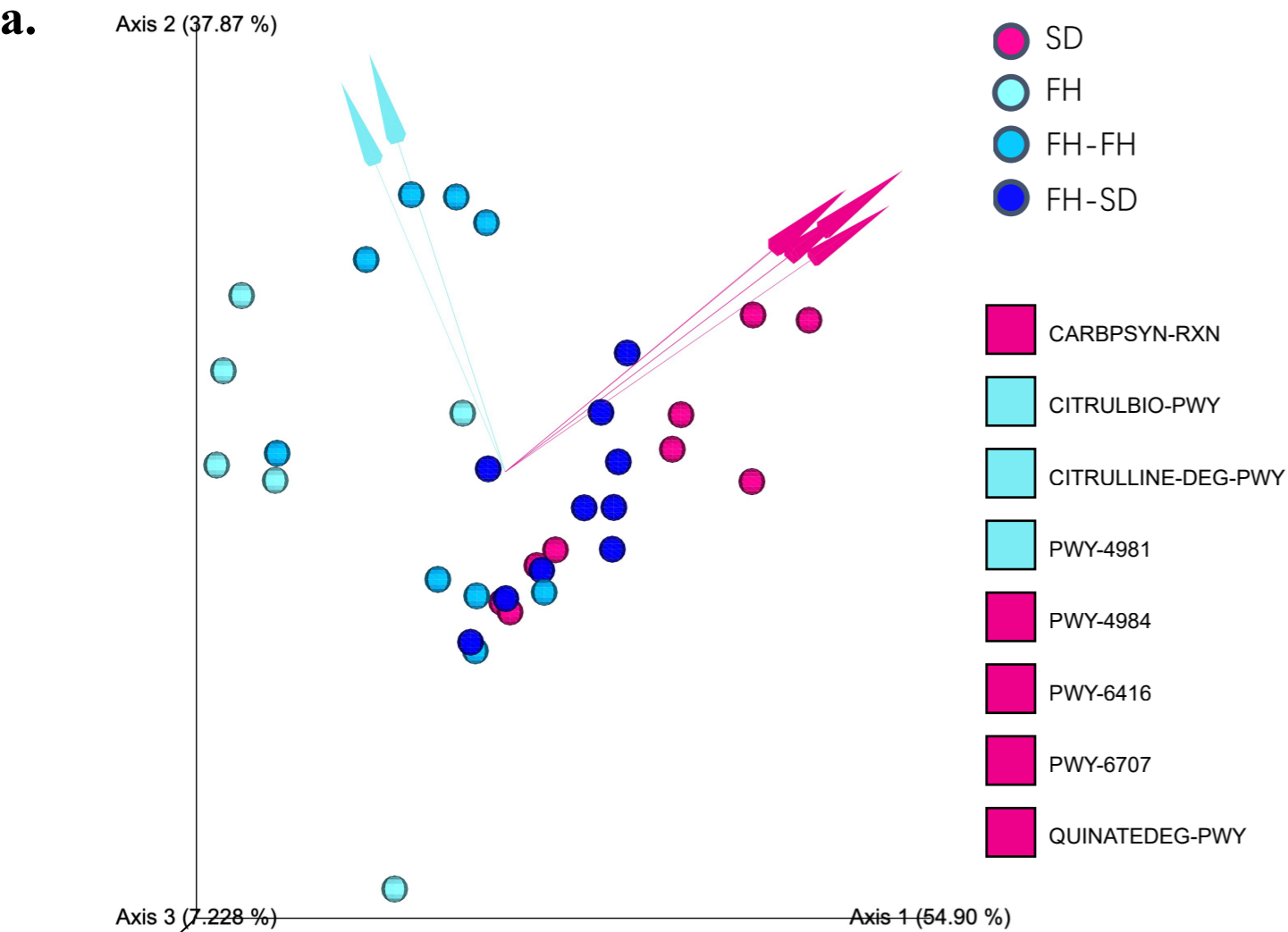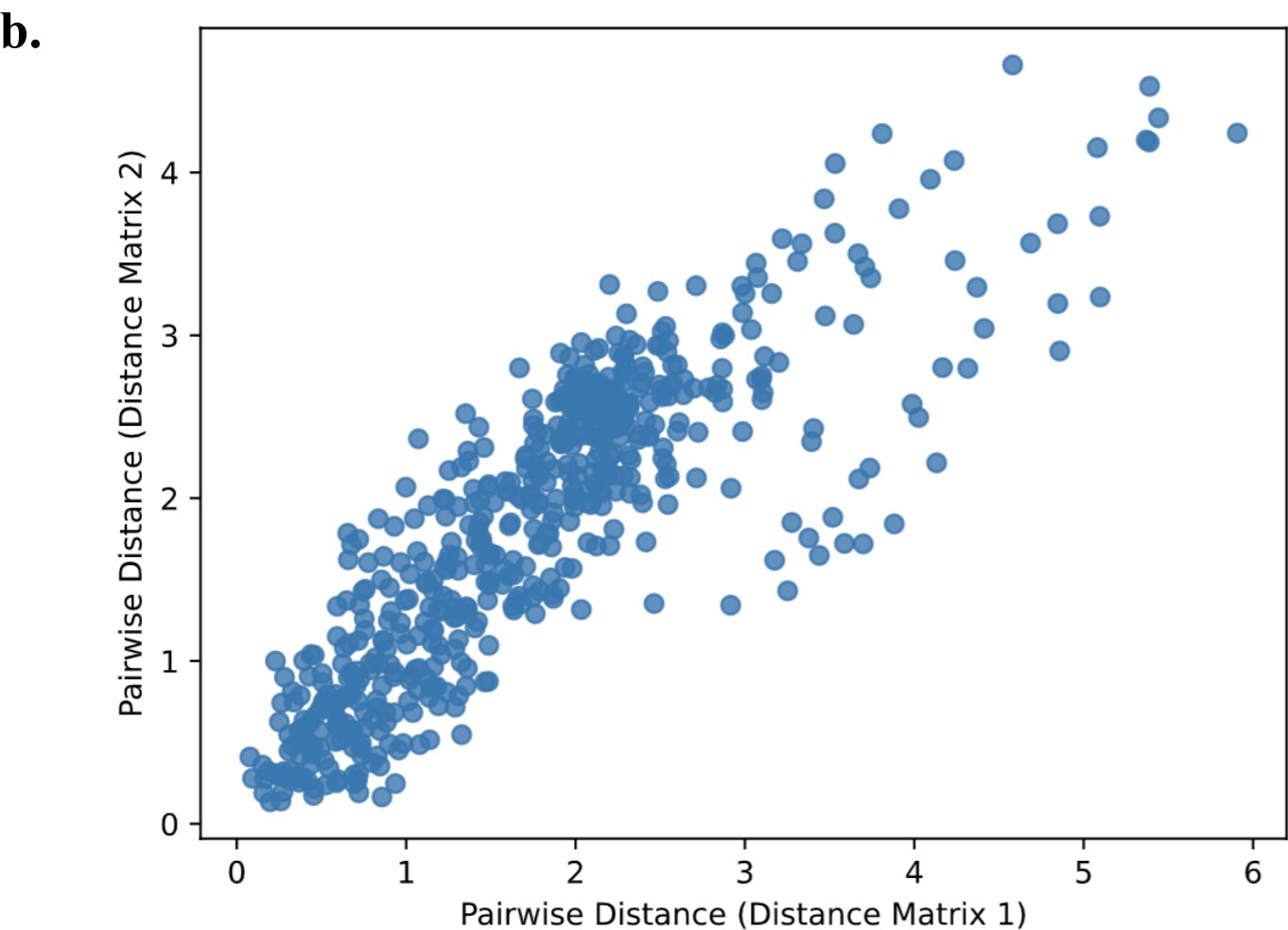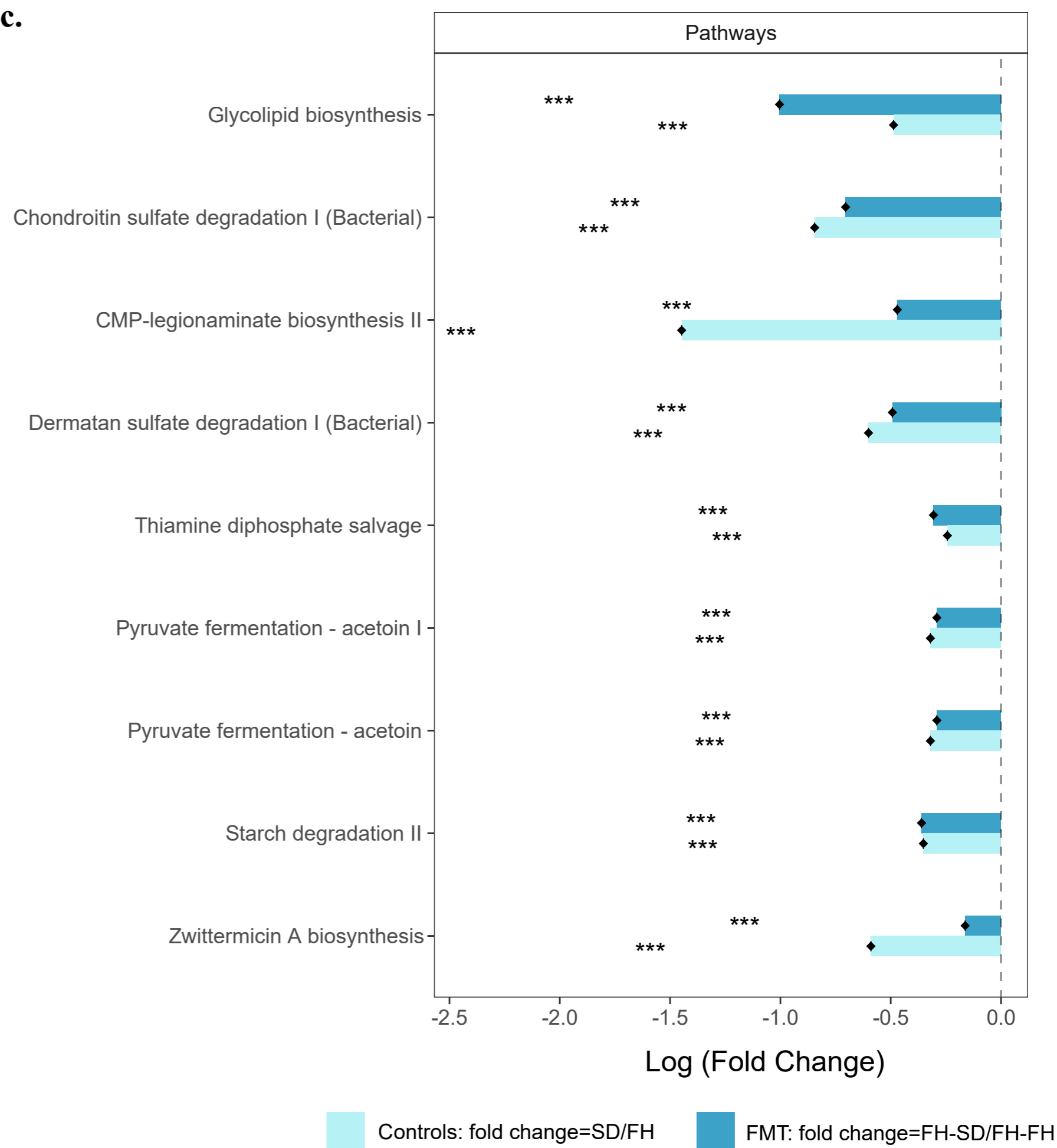

Supplement: FIG S3 [file msystems.00218-22-s0003.pdf]

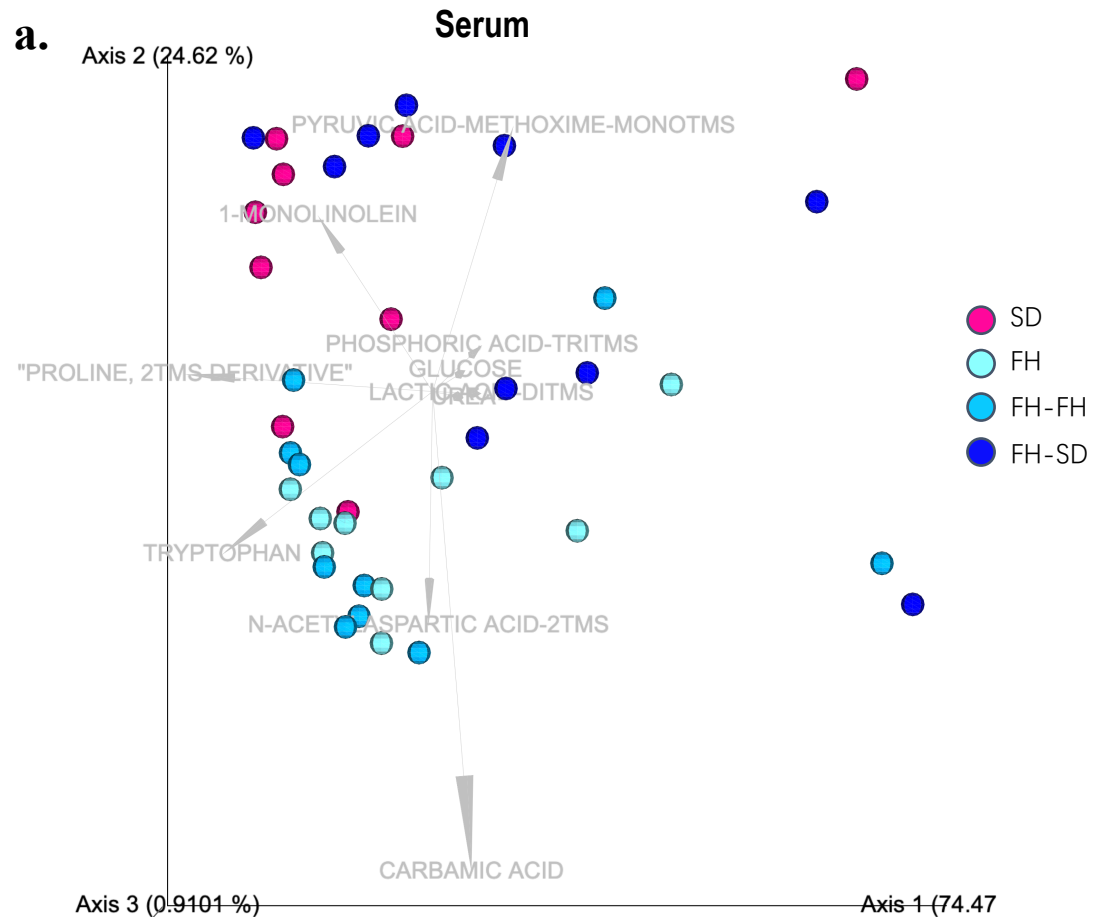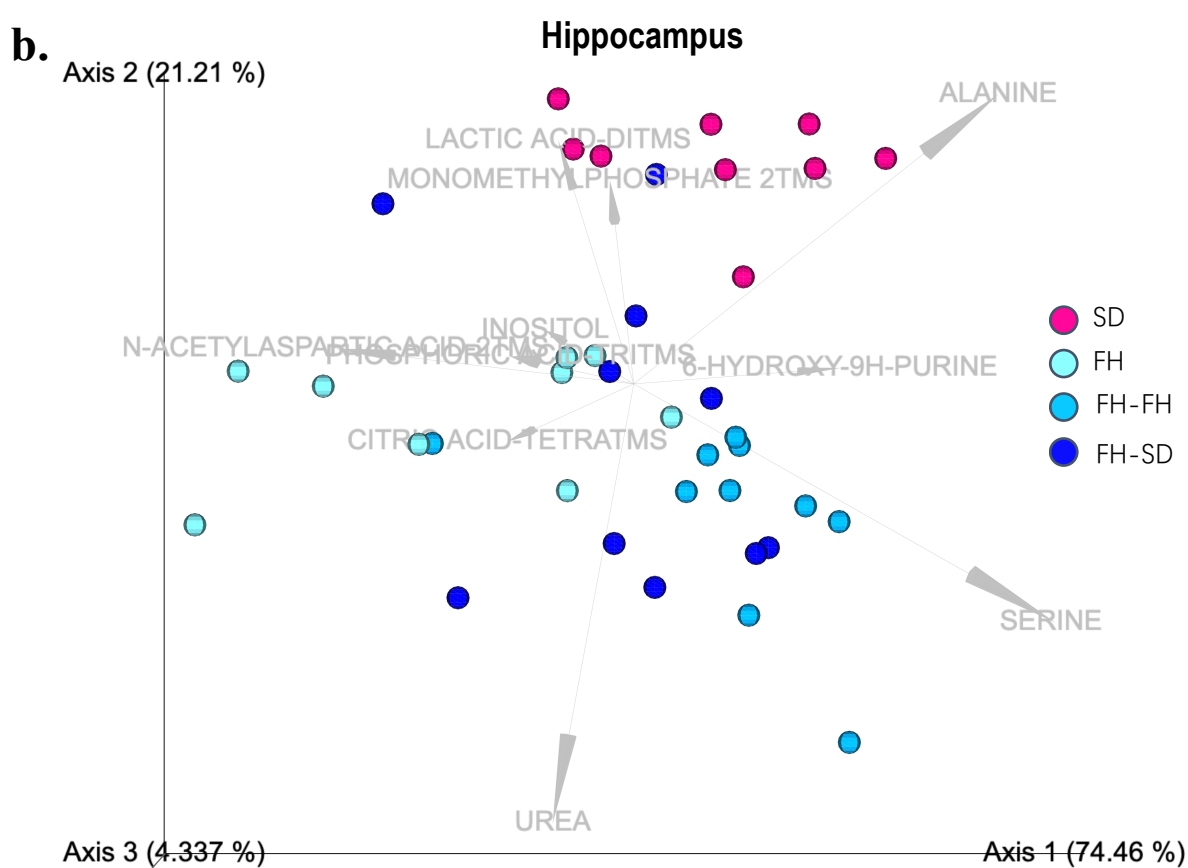

Supplement: FIG S4 [file msystems.00218-22-s0004.pdf]

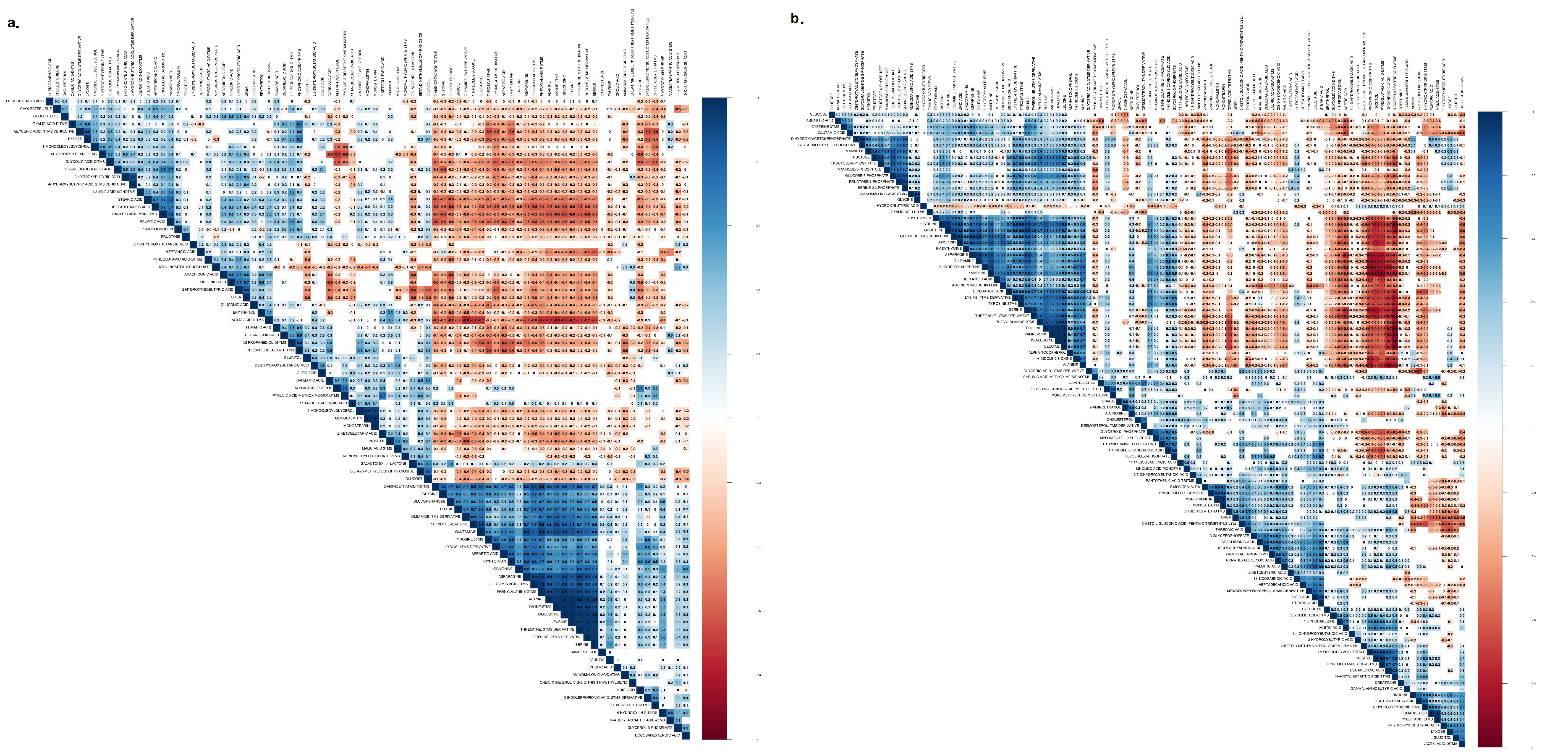

Supplement: FIG S5 [file msystems.00218-22-s0005.jpg]

a.

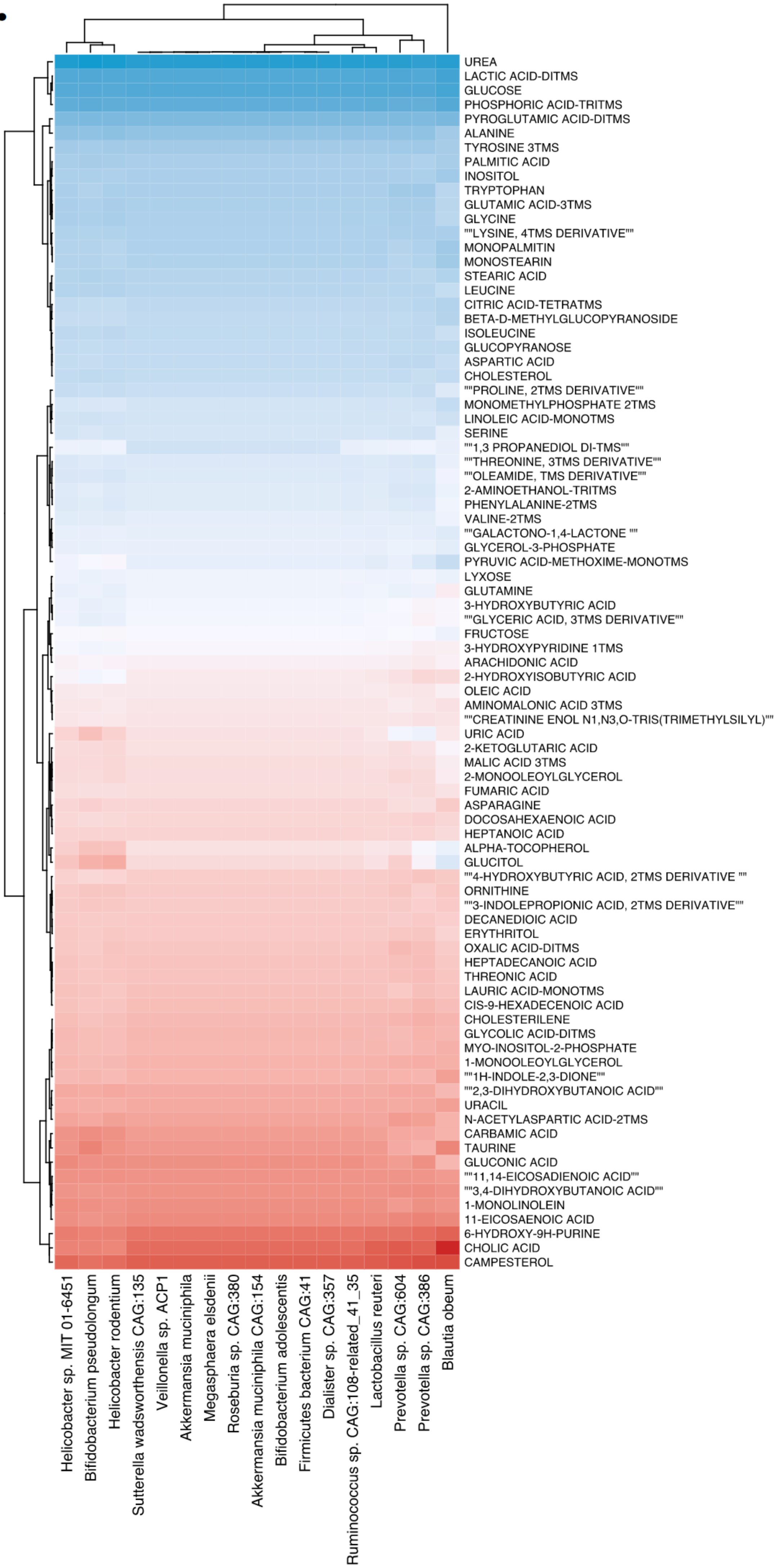

Conditional Probability

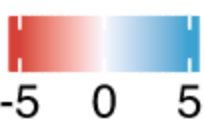

b.

Pseudo Q-squared: 0.350289

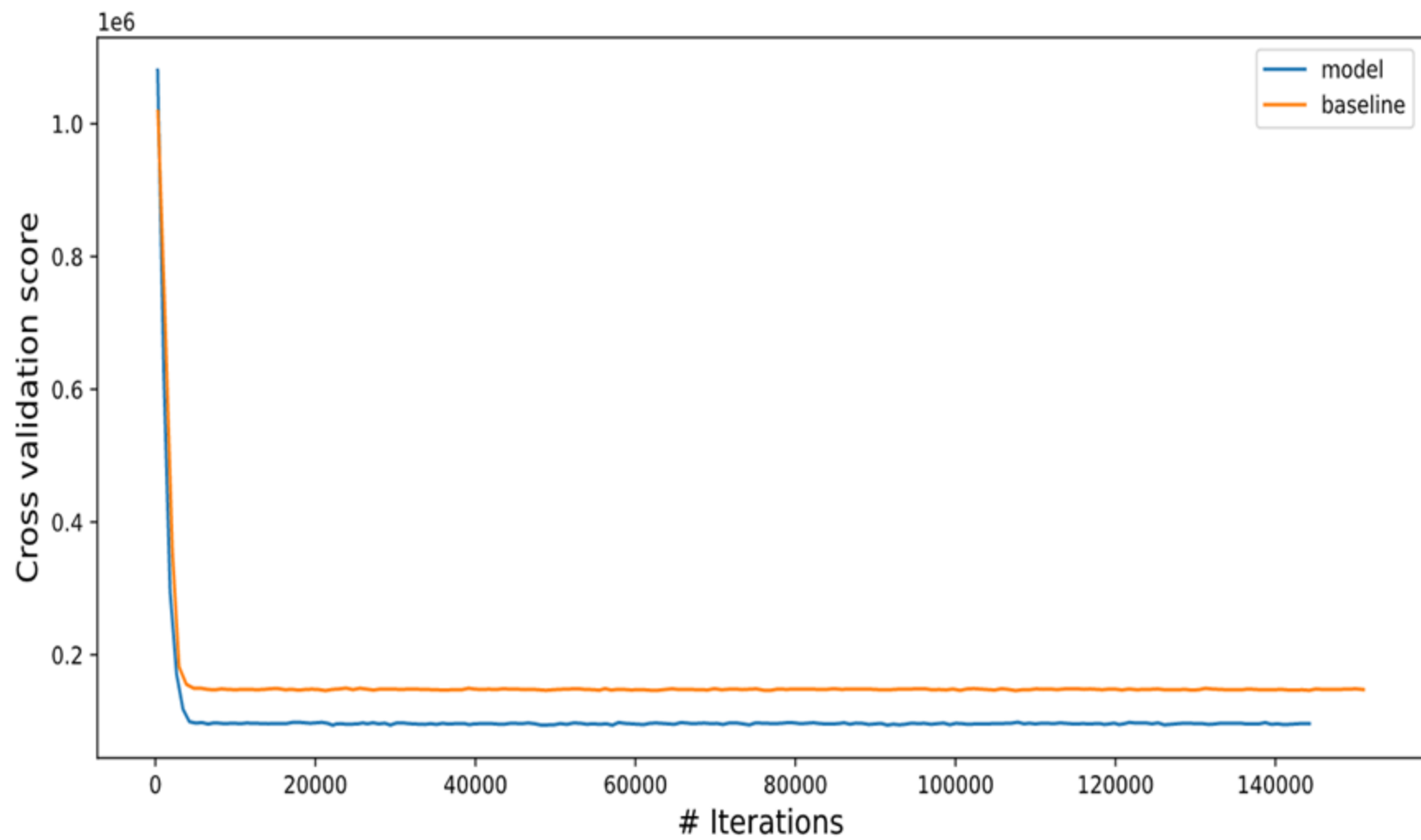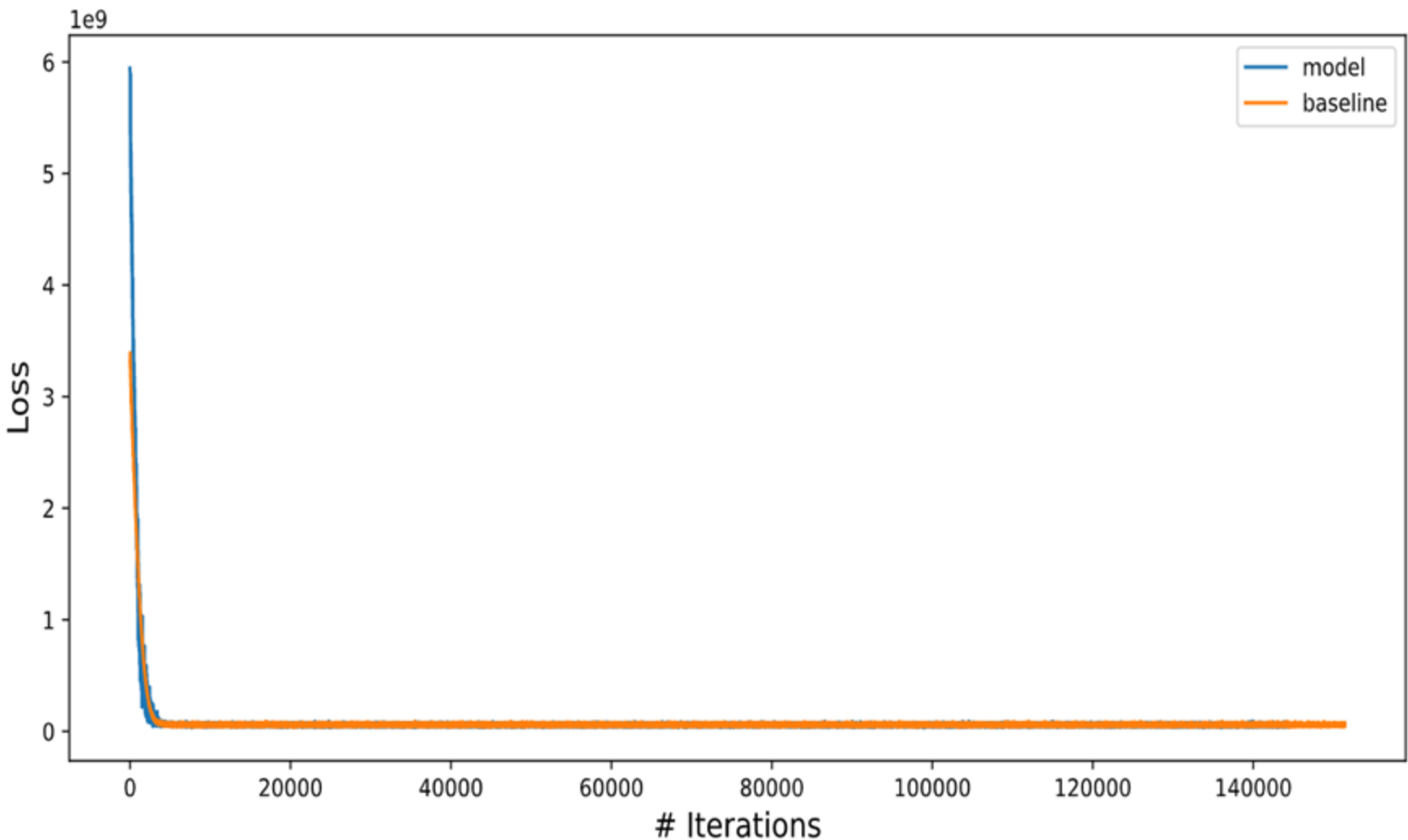

Supplement: FIG S7 [file msystems.00218-22-s0007.pdf]
